# Treatment of Fresh Meat, Fish and Products Thereof with Cold Atmospheric Plasma to Inactivate Microbial Pathogens and Extend Shelf Life

**DOI:** 10.3390/foods11233865

**Published:** 2022-11-30

**Authors:** Peter Paulsen, Isabella Csadek, Alexandra Bauer, Kathrine H. Bak, Pia Weidinger, Karin Schwaiger, Norbert Nowotny, James Walsh, Emilio Martines, Frans J. M. Smulders

**Affiliations:** 1 Unit of Food Hygiene and Technology, Institute of Food Safety, Food Technology and Veterinary Public Health, University of Veterinary Medicine, Veterinaerplatz 1, 1210 Vienna, Austria; 2 Canfelis, Kienmayergasse 5, 1140 Vienna, Austria; 3 Viral Zoonoses, Emerging and Vector-Borne Infections Group, Institute of Virology, University of Veterinary Medicine, Veterinaerplatz 1, 1210 Vienna, Austria; 4 Department of Basic Medical Sciences, College of Medicine, Mohammed Bin Rashid University of Medicine and Health Sciences, Dubai P.O. Box 505055, United Arab Emirates; 5 Centre for Plasma Microbiology, University of Liverpool, Liverpool L69 3BX, UK; 6 Department of Physics “G. Occhialini”, University of Milano—Bicocca, Piazza della Scienza 3, 20126 Milano, Italy

**Keywords:** cold atmospheric plasma, antimicrobial effects, physical-chemical properties, foodborne pathogens management, longitudinally integrated safety assurance, shelf-life extension

## Abstract

Assuring the safety of muscle foods and seafood is based on prerequisites and specific measures targeted against defined hazards. This concept is augmented by ‘interventions’, which are chemical or physical treatments, not genuinely part of the production process, but rather implemented in the framework of a safety assurance system. The present paper focuses on ‘Cold Atmospheric pressure Plasma’ (CAP) as an emerging non-thermal intervention for microbial decontamination. Over the past decade, a vast number of studies have explored the antimicrobial potential of different CAP systems against a plethora of different foodborne microorganisms. This contribution aims at providing a comprehensive reference and appraisal of the latest literature in the area, with a specific focus on the use of CAP for the treatment of fresh meat, fish and associated products to inactivate microbial pathogens and extend shelf life. Aspects such as changes to organoleptic and nutritional value alongside other matrix effects are considered, so as to provide the reader with a clear insight into the advantages and disadvantages of CAP-based decontamination strategies.

## 1. Introduction

In the food production sector, ‘shelf life’ is one of the most essential quality parameters. Even when microbial contamination and subsequent growth of pathogenic organisms are successfully counteracted, microbial and/or chemical spoilage will cause foods of animal origin to be withdrawn from the market. The latter is one of the main worries of the United Nations (UN), as such represents the main reason for food waste. Approximately one-third of the world’s foods of animal origin is lost through waste and this markedly reduces food security [1]. According to estimates of the UN’s Food and Agricultural Organisation (FAO), published in 2015, it results in approximately USD 940 billion per year in economic losses. It also results in significant environmental impacts. For example, food loss and waste are responsible for 8% of the world’s greenhouse gas emissions [2]. In fact, if food loss and waste were contained to one country, that country would be the world’s third-largest emitter after the United States and China [1].

Arguably, food security is not only dependent on minimising food waste, but also on the enhancement of the efficacy of meat production. Thus, farm-animal species play crucial roles in satisfying demands for meat on a global scale, and environmental as well as genetic factors [3] need to be optimised. In particular, one of the important aims is to increase skeletal muscle growth in farm animals [4,5]. The enhancement of muscle development and growth is crucial to meet consumers’ demands for meat [5,6].

The veterinary medical curriculum includes enough elements of biology and physiology to allow graduates to function as ‘doctors’, yet many of them end up working in the food industry as ‘veterinary public health’ (VPH) professionals. These have the legal responsibility to remain aware of the latest technologies and techniques applied by industry to assure their products are safe, nutritious and have the desirable physical-chemical and sensory properties to appeal to the customer. Over the past decades, VPH officials have gradually shifted their attention from ‘end-product oriented inspection’ towards ‘longitudinally integrated safety assurance’ (LISA; [7]) and as health officials they concentrate on assuring the absence of pathogenic microorganisms in foods of animal origin as evidence for ‘quality’. However, in the current political climate, public health authorities need to assure that besides ‘food safety’ (the first, apparently most significant parameter), also ‘food security’ and ‘sustainability’ issues are adequately addressed. ‘Food security’ has been defined by the UN’s FAO as: ‘*assuring that all people, at all times, have physical and economic access to sufficient, safe and nutritious food, that meets their dietary needs and food preferences for an active and healthy life*’, and ‘sustainability’ as: ‘*meeting the needs of the present without compromising the ability of the future generations to meet their own needs*’ [8]. The paramount importance of food security was emphasised in the UN World Commission on Environment and Development Report in 1987 [9].

Concentrating on safety, security and sustainability is ‘part and parcel’ of the EU’s current pathogen management strategy, which has been summarised in its May 2020 strategy paper ‘From Farm to Fork’ [10]. For this contribution, this means that the authors take an approach beyond merely judging the antimicrobial efficacy of risk management strategies against pathogens, but rather additionally consider effects on variables such as shelf life and physical-chemical and sensory attributes. In particular, the exposure of foods to reactive species produced in an ionised gas—‘plasma’—and their effects on microbial contaminants and on the food matrix (in terms of sensory quality and alterations of proteins and lipids) will be discussed. To this end, we provide (i) an introduction on the composition and generation of plasma, and (ii) an overview of the application of plasma technology for the microbial decontamination of selected food commodities of animal origin, with (iii) special consideration of the effects of plasma on the sensory quality of meat products, in particular those related to oxidative reactions. The antibacterial potential of the application of plasma on meat-based products was emphasised recently [11], but the oxidation of lipids [12] and proteins [11] has been identified as a potential drawback. Although protein modification can have positive side effects (e.g., improving gelling quality, [13]), protein oxidation has been identified as a major cause for quality loss in muscle-based foods [14,15].

## 2. What Is ‘Cold Atmospheric Plasma’ and How Is It Generated?

### 2.1. Generation of Plasma

The term ’plasma’, originally coined by Nobel prize winner Irving Langmuir, designates in physical sciences a gas where a fraction of the particles is ionised; that is, stripped of one electron and converted into an electron–ion couple. The plasma state, which is considered as the fourth state of matter, is thus a mixture of electrons, ions and neutral particles [16]. The fraction of charged to total particles, called ‘degree of ionization’, is a function of several factors, among which the most notable one is the power density used to produce the plasma, and can range from very low values (weakly ionised plasma) to 1 (fully ionised plasma). The plasmas typically used for the treatment of food are considered weakly ionised, so most of the gas particles are electrically neutral.

In almost all practical situations, plasma is produced by the application of an electric field. The electric field accelerates free electrons to the energy required to ionise neutral atoms and molecules in the gas (typically between 5 and 25 eV). This is, however, opposed by collisions that electrons undergo in their motion through the gas: in particular, inelastic collisions cause electrons to lose energy, which is transferred to the neutral gas in the form of the excitation of bound electrons to higher energy levels, or the excitation of the rotation or vibrational states of molecules. Incidentally, the emission of photons when the excited bound electrons return to their original state is the source of visible light, which gives plasmas their typical glowing appearance, and can also be a source of biocidal ultraviolet (UV) radiation. Given this balance between acceleration and collisional energy loss, a minimum applied voltage is required for plasma ignition, a phenomenon called ‘breakdown’, because the presence of free charged particles converts the previously dielectric neutral gas into an electrical conductor.

To achieve breakdown, it is required that the few free electrons naturally present in the gas are increased in number. This occurs through a process, by which collisions between neutral species and sufficiently energetic electrons result in ionisation events, causing the number of free electrons in the gas to grow exponentially. This process is known as an ‘electron avalanche’. Subsequent processes strongly depend on the manner in which the electrical energy is applied. In the case of a stationary (direct current, DC) or slowly varying electric field, the positive ions produced in ionisation events are accelerated towards the negative electrode, called the cathode (assuming that the electric field is obtained by applying a potential difference between two electrodes), and have a certain probability of extracting an electron called the ‘secondary electron’ from its surface. When this process is sufficiently intense to provide a new electron for each electron lost to the anode, the process becomes self-sustaining. A second approach is to vary the electric field fast enough that electrons perform an oscillation with an amplitude smaller than the electrode gap. In this case, there is no electron flux to the anode, and each electron can produce others in its oscillatory motion (the electron number does not grow indefinitely because of diffusion losses). For typical system sizes, ranging from mm to cm, the required oscillation frequency is in the order of a few MHz or larger: this defines the so-called ‘radio frequency (RF) plasmas’, or, when frequencies are in the GHz range, ‘microwave plasmas’.

Since the electric field readily transfers energy to electrons, they typically have high temperatures, in the order of 1 eV or higher (in plasma physics, temperatures are given through their equivalent mean kinetic energy: 1 eV corresponds to a temperature of 11,600 K). Still, unless very high power is used, and the plasma is very well confined (for example, by magnetic fields, as is the case in thermonuclear fusion studies), most electrons do not have the time to transfer their energy to the ions and to the neutral gas, which thus remain at relatively low temperatures. In this case, which is the one of interest in the following, the plasma can be described as ‘non-thermal’. If the ions and the neutral gas remain at, or very near room temperature, the notion of ‘cold plasma’ arises. This is exactly the concept of interest here since the use of plasma for food decontamination must avoid thermal effects. For a detailed description of the underlying principles of plasma generation and applications in materials modification, the reader is referred to Lieberman and Lichtenberg [17].

The interest of plasmas in the context of disinfection and decontamination (as in many other fields) stems from the fact that the free hot electrons shift chemical equilibria, giving rise to a wealth of reactive species, which interact in a destructive way with microbes. In particular, reactive oxygen and nitrogen species (ROS and RNS) are of relevance for this application, due to their interaction with the cell membrane [18,19]. Furthermore, the plasma is responsible for generating other agents, namely UV radiation, intense electric fields and charged species, which may also play a role in the decontamination process [20,21].

### 2.2. Low Pressure Plasma vs. Atmospheric-Pressure Plasma

Plasmas are most easily produced at low pressure (several orders of magnitude below atmospheric pressure) because the electrons do not undergo too many collisions and are easily accelerated to the energy required for ionisation. This results in a reduced breakdown voltage, typically of the order of a few hundred volts. However, low-pressure plasmas are not well suited for food treatment. On the contrary, generating plasma at atmospheric pressure requires higher voltages, typically a few kV [22]. Consequently, the requirement of keeping the neutral component at or near room temperature results in the need to limit the current achieved after breakdown, otherwise it is very easy to obtain high power levels. There are different solutions for this problem, which can be summarised into two main approaches. One is the dielectric barrier discharge (DBD), where the high-voltage electrode is separated from the grounded electrode by at least one layer of dielectric material. After the breakdown, charge quickly accumulates on the dielectric surface, and extinguishes the current. These devices are typically operated at a frequency of a few kHz, although lower frequencies can be used, or with pulsed voltages [23]. The second one is the use of radio frequency (RF) voltage, which changes polarity so quickly that the peak current is limited. In the context of plasma food treatment, RF is seldom used, as such plasmas are typically ‘hotter’ than those generated using the DBD approach.

### 2.3. Application of Atmospheric-Pressure Plasma to Tissues, Foods and Food Contact Surfaces

The potential of atmospheric-pressure plasma to inactivate bacteria and other pathogens has been known for a long time [24]. However, only in the last twenty years has the development of low-power plasma sources enabled the treatment of organic substrates without thermal damage, leading to its use in the emerging discipline of ‘plasma medicine’ [25]. This includes not only disinfection [26], but also the use of the plasma-produced chemical species to manipulate cellular processes or structures [19] giving rise to therapeutic effects. For example, the stimulation of wound healing is a well-studied process [27,28,29,30], which has found application also in the context of veterinary medicine [31]. A field where the use of plasma-based decontamination may prove to be a game changer is that of food decontamination [32]. A particular mode of plasma generation has been studied more recently, with a view to assess its suitability as a means of antimicrobial intervention during food production [33]. The scientific principles on which the generation of reactive gas species is based, and the modes of antimicrobial action of cold atmospheric plasma (CAP) when applied to various foods of animal origin, with special reference to meat and meat products, have been reviewed recently [34].

It is essential to realise that CAP exerts its antimicrobial action primarily on the surface of a treated food item. Hence, bacterial (or viral) surface contamination resulting from slaughter and subsequent processing could therefore—at least partly—be inactivated before further processing/packaging. Obviously, the chosen method of applying plasma determines the antimicrobial efficacy of exposing microbial contaminants to plasma.

The specific mix of reactive species produced by a plasma source depends on several interlinked factors. First is the gas used for the process, which is typically either air or a noble gas (helium or argon) mixed with small fractions of air or air constituents. Second is the amplitude, frequency and waveform of the voltage used to produce the plasma, the applied power and the gas flow (if applicable). For example, in the case of an air DBD, the prevalence of ROS or RNS will be dictated by the power level [35]. As another example, humidity will affect ozone production [36], and this may lead to the loss of bactericidal effect [37]. The closer the target is to the plasma source, the shorter the time needed for reactive species to reach the target. In settings with a distance between electrodes and the sample, the presence of long-lived radicals such as NO_2_, O_3_ and N_2_O is an important factor co-determining the array of reactive compounds.

**Figure 1:**
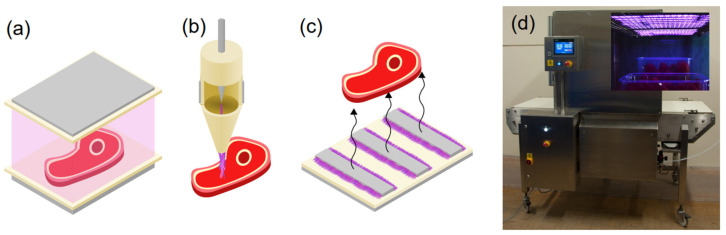
Typical DBD systems used for the treatment of food products: (**a**) direct-contact parallel plate reactor, (**b**) direct-contact plasma jet, (**c**) indirect contact surface barrier discharge and (**d**) pilot-scale surface barrier discharge system developed at the University of Liverpool.

In recent years, a plethora of different plasma systems have been developed for the decontamination of food contact surfaces [38] and food products [39]. Typically, but not exclusively, these prototype systems have been based on the DBD family of discharges. Two distinct modes of application have arisen, and the first involves plasma interacting directly with food products, typically achieved by placing the product between the electrodes of a parallel plate reactor or in the effluent of a plasma jet (Figure 1a,b, respectively). Direct contact systems are highly efficient as reactive and short-lived chemical species, such as O, N and OH, directly impinge on, and interact with, the food matrix. Despite their efficiency, a direct contact between plasma and food poses a number of technical challenges; for example, the plasma characteristics are inevitably and inextricably linked to the electrical characteristics of the food product, a situation that can compromise repeatability. Another challenge relates to the complexity of the discharge chemistry reaching the product and its impact on the food matrix, a process that could potentially involve over 1000 complex biochemical reactions. Without a clear understanding of the underpinning processes that give rise to the intriguing antimicrobial effects associated with plasma treatment, the regulatory approval necessary for the commercial application of direct-contact plasma technology may not be forthcoming.

The second mode of application relates to the indirect exposure of food products to plasma, typically achieved by generating plasma in close proximity to a product and relying on the diffusion and/or convection of chemical species to its surface (Figure 1c). In this scenario, short-lived chemical species react before reaching the food product, yielding a number of longer-lived intermediaries; for example, in the case of air plasma O_3_, NO, N_2_O and NO_2_ [40]. Due to the absence of highly reactive chemical species, indirect approaches are often considered less effective for microbial inactivation compared to their direct-contact counterparts. Conversely, a vast reduction in the variety of chemical species reaching the product is conducive when attempting to elucidate the underpinning mode of action. A further benefit of many indirect treatment approaches is their ability to be easily scaled to cover large areas (Figure 1d), and they remain unaffected by the electrical properties of the food product, enhancing repeatability.

### 2.4. In-Package Cold Plasma Treatment

Direct exposure to CAP can also be achieved for already packed products. In this case, the packaging material itself is used as the dielectric barrier and external electrodes are used to apply a high voltage, resulting in plasma formation directly within the sealed pack. This efficacy of ‘in-pack’ plasma treatment of foods has been demonstrated against foodborne pathogens [41,42] and spoilage microorganisms to extend the shelf life of end products [43,44,45,46,47]. As products are already sealed within the package prior to plasma disinfection, there is little opportunity for further contamination, which is considered a major advantage of the approach.

A drawback of the approach is the requirement that the packaging material can withstand plasma treatment without degradation, which could potentially contaminate foods sealed within. Very few studies have considered the impact of in-package plasma on the packaging material, or how the material influences the production of plasma species [46,47]. Previous studies have shown that the physical-chemical and microbiological condition of fresh beef, packaged in a polyethylene–polyamide–polyethylene (PE/PA/PE) film after it had been inoculated with *S. aureus*, *L. monocytogenes* and *E. coli*, is in no way affected by subjecting it to treatment with atmospheric-pressure cold plasma ([48]; some details in Section 3).

Using in-package plasma treatment (2 to 60 s), statistically significant reductions (0.8–1.6 log cycles) in *Listeria innocua* contamination of ‘Bresaola’ (a dried, ready-to-eat beef ham product) were recorded [49]. Using ‘dielectric barrier’ electrodes and air as gas, a 3 min plasma treatment of packaged chicken cubes allowed reductions in *Salmonella*, *E. coli* O157:H7 and *L. monocytogenes* of up to 3.7 log [50]. Similar findings were recorded by Jayasena et al. [51], who established antimicrobial effects of up to 2.6 log *S. typhimurium* and *L. monocytogenes* in PE-packaged beef and pork using a DBD system.

British studies have shown that at retail level a sizeable portion of the cross-contamination of fresh meat occurs via both the external and internal surfaces of the packaging film [52]. Until the CAP exposure of food and food products has gained the necessary regulatory approval, the application of CAP to inactivate pathogens on the external surfaces of packaging materials is a viable way forward, provided one can demonstrate that the packaging matrix is not breached by cold plasma and hence that the packaged product does not have to be classified as a ‘novel food’ according to EU Regulation 2015/2283 [53]. According to the latter regulation, any lasting change incurred beyond what could be expected naturally as a result of plasma exposure would require ‘*novel food*‘ certification [53]. As regards plasma generated from ambient air, it is debatable if the action of ROS and RNS would qualify plasma-treated food as ‘novel food‘ (i.e., food with intentionally modified molecular structures that were not present in foods within the Union before 15th May 1997, or ‘*food resulting from a production process not used for food production within the Union before 15 May 1997, which gives rise to significant changes in the composition or structure of a food, affecting its nutritional value, metabolism or level of undesirable substances*’; Article 3, paragraphs (i) and (vii) of Regulation (EU) 2015/2283; [53]). European partner countries initiated a COST project (013/20) with the purpose of creating a database that will ultimately allow ‘understanding plasma’s most important processes including aspects of (Novel food) legislation, energy consumption, food safety and quality’. However, provided a CAP treatment does NOT cause a lasting change, it can be considered a processing aid and can be applied without extra labelling or consumer information [54]. A case-by-case evaluation of plasma-matrix combinations has been suggested [55]. In 2017, Ekezie et al. [56] reported that CAP had not been implemented in food industry settings because of uncertainties about matrix modifications and subsequent legal issues. In the last years, this knowledge gap has been at least partially filled; see the following sections.

## 3. Effects of CAP Treatment of Fresh Meat and Meat Products

### 3.1. Fresh Meat

Microbial contamination of meat occurs at slaughter and numerous contamination scenarios are possible along the fresh-meat chain [57,58]. Control of such contamination is essential to prevent food from becoming ‘unsafe’, i.e., either hazardous to human health or unfit for human consumption due to spoilage (Regulation (EC) No 178/2002) [59]. The prospects and limitations of ‘Good Hygiene Practice’ and, more specifically, of ‘Hazard Analysis Critical Control Point’ systems in safeguarding fresh meat have been extensively discussed [60,61] and the usefulness of interventions as additional tools has been proposed and studied [62,63,64,65,66,67,68].

Typically, such methods would be preservation or processing, but these usually alter product appearance or other characteristics. Thus, the array of intervention methods for fresh meat is rather limited. Treatment with cold or hot water or with steam and dilute organic acids may be applied by rinsing, spraying or immersion, with no or negligible effects on the appearance of fresh meat and without leaving residues [69,70,71,72,73,74,75,76]). Since nearly a decade, the application of dilute lactic acid has been allowed in the EU, albeit only as part of the pre-chill treatment of beef carcasses (Commission Regulation (EU) No 101/2013) [77].

The possibilities of exposing meat surfaces to further decontaminating treatments that aim to eliminate both pathogens and spoilage flora has remained a relevant topic. Treatment with CAP has been suggested as a promising option [33,48]. Three major questions need to be addressed, i.e., ‘What level of microbial reduction can be achieved?’, ‘Are there significant ‘side-effects’ on the meat matrix’ and, in in-pack exposure settings: ‘How do packaging material and headspace in the package influence the effect of CAP?’ The latter issue has already been addressed in this paper (see Section 2.4). A number of studies have demonstrated the antibacterial activity of CAP on meat surfaces, considering the possibility of changes in the meat matrix due to lipid oxidation, protein denaturation and the state of haem pigments.

The magnitude of the reduction in contaminant bacteria by CAP technology is influenced by the nature and abundance of plasma gas species, which, in turn, is different depending on the medium in which the plasma is generated and on the way the gas species get in contact with the food matrix, e.g., direct exposure or circulating gases or liquids. Thus, it has been suggested to consider key parameters when comparing the outcomes of different studies [11]. Since our aim was not to identify the ‘best treatment protocol’, we refrained from presenting all experimental details in the various studies discussed in this paper. Still, the matrix in which plasma was generated (gases or liquids) and exposure conditions are given.

There are also differences in susceptibility between bacterial genera and the physical state of the meat samples (e.g., chilled or deep-frozen). For example, Choi et al. [78] contaminated samples of frozen and fresh pork with *L. monocytogenes* and *E. coli*. Samples were then exposed to CAP generated by a corona discharge plasma system (20 kV DC, 58 kHz) with a fan delivering air to the plasma source. A 120 s exposure to CAP effectuated a reduction in the numbers of *E. coli* by about 1.6 log CFU in fresh, but significantly more (about 2.7 log) in frozen pork. Reductions in *Listeria* were ca. 1.1 log CFU, with no significant difference between chilled and deep-frozen pork. Arguably, all contaminated surfaces must be exposed to CAP to achieve optimum results. Thus, Yong et al. [79] found that *E. coli* on raw chicken breast was reduced by 1.14 log CFU when one side was exposed to CAP (generated from an O_2_:N_2_ mix) for 5 min, but by 1.44 log CFU, when both sides of the fillet were exposed for 2.5 min each.

Depending on the distance from the plasma source to the target surface, either a wide array of reactive gas species may reach and interact with the target, or only long-lived species may arrive; see Section 2.3. The gas species will react with compounds of the cell wall (including the cell membrane) and with cytoplasmic components and nucleic acids [80]. Most compounds (with the exception of cell wall components) are not exclusive for bacteria, but also prevail in eukaryotic cells. Thus, it can be expected that the majority of the arriving reactive species will react with the more abundant food matrix rather than with the bacterial cells. Since penetration depth is low and the underlying meat parts will act as buffers, the ‘average’ immediate effect on a piece of meat with several cm diameter can be expected to be negligible. A somewhat different situation may exist with respect to triggering the auto-oxidation of lipids. Since (apart from water) protein and lipids are the major constituents of meat [81], numerous studies have focused on the consequences of CAP exposure to lipids [12,82] and proteins, including haem proteins [83]. In addition to chemical tests, colour measurement has proven to be able to assess sarcoplasmic denaturation (indicated by an increase in L*) [48] or oxygenation or oxidation of myo- and haemoglobin (indicated as changes in a* values). Finally, nitrate generated in water exposed to CAP can be ultimately reduced to NO, resulting in the development of NO-myoglobin, which, after heat treatment or other type of denaturation, turns into the pink NO-haemochrome [83]. Notably, in water exposed to CAP generated in air, nitrate will accumulate, which would allow the use of such plasma-activated water as a curing agent (see Section 4). RNS are typically produced in atmospheric pressure air plasmas with a high-power density at higher voltage, whereas plasma generated at lower powers are dominated by ROS [52]. In principle, voltage adjustment would allow fine-tuning if antibacterial action with the side-effect of oxidation (low power) or curing (high power)—with an antioxidative side-effect—is aimed at [84,85].

Bauer et al. [48] studied the effects of plasma treatment of packaged fresh beef. Vacuum packaged and non-packaged beef longissimus samples were treated with CAP (generated from ambient air, at different powers) over a 10-day period of vacuum, and a subsequent 3-day period of aerobic storage. It is important to realise that their approach was fundamentally different from treating foods ‘in-pack’ (i.e., after packaging) as described above under Section 2.4. Exposure of ‘non-covered’ beef samples to high-power CAP conditions resulted in increased a*, b*, Chroma and Hue values, but CAP treatment of packaged loins did not impact colour (L*, a*, b*, Chroma, Hue), lipid peroxidation, sarcoplasmic protein denaturation, nitrate/nitrite uptake or myoglobin isoform distribution [48]. Colour values measured after 3 days of aerobic storage following un-packaging (i.e., at 20 days post-mortem) were similar and all compliant with consumer acceptability standards. Exposure to CAP of the polyamide-polyethylene packaging film inoculated with *Staphylococcus aureus*, *Listeria monocytogenes* and two *Escherichia coli* strains resulted in a >2 log reduction without affecting the integrity of the packaging matrix. Results indicate that CAP can reduce microbial numbers on the surfaces of beef packages without affecting the characteristics of the packaged beef. 

### 3.2. Meat Products

The potential use of CAP as a decontamination technology has also been studied in meat products. This included dried—‘jerky style’ [84,85,86]—products as well as dried-cured meat products such as dry ham [37,49].

In cooked/cured meat products, CAP has been used mainly as a curing agent—either by direct curing or by making use of plasma-treated water (PTW)—through the formation of reactive nitrogen species (RNS) when relying on N_2_ as part of the carrier gas mixture leading to the formation of nitrite, and hence producing a curing effect [87,88,89,90,91].

Cooked/cured meat products are generally microbiologically safe due to the combination of heat and nitrite [92,93]. However, post-processing handling such as cutting, slicing and packaging may lead to recontamination of the surface [94,95]. Thus, it is advantageous that CAP is effective on the product surface due to the nature of plasma [48]. As regards the post-processing and pre-packaging contamination of ready-to-eat cooked meats, *Listeria monocytogenes* is the pathogen of concern [96], the more so as pH and water activity are often not low enough [97] to prevent the multiplication of *L. monocytogenes* during the shelf life of the product. In a study on typical Austrian cooked ready-to-eat meat products [98], this issue was addressed in detail, and a decision tool was developed to estimate to what extent pH or water activity of a given product need to be adjusted, albeit the authors concluded that there are limited possibilities to do so without altering sensory product characteristics and impacting consumers´ acceptance. This issue has been studied in detail in typical Austrian cured-cooked meats, see Csadek et al. [99], with respect to *Listeria* and *E. coli* and to colour changes after exposure to CAP generated from ambient air. The authors found that *E. coli* was more readily reduced than *Listeria*. It has been speculated that Gram-negative bacteria (*E. coli*) are more susceptible for CAP than are Gram-positives (*Listeria*), since the membrane lipids in Gram-negative organisms are directly exposed to CAP molecules, in particular ozone, whereas the cell wall of Gram-positive organisms would protect the cell membrane. However, experimental data are inconclusive [37,39,48]. Differences were also observed between the high and low power settings of the CAP device, but also between similar products from different manufacturers. Since the composition of the samples was—according to the information provided on the label—practically the same, it remains to be explored why different results were obtained.

## 4. Effects of CAP Treatment of Aquatic Foods of Animal Origin: Review of Recent Model Experiments

‘Aquatic food’ means food grown in or harvested from water (including all types of fish, reptiles and amphibians) and mixtures containing aquatic foods and synthetic foods, such as surimi. It is important to realise that the terms ‘aquatic foods’ and ‘seafoods’ are not necessarily considered to be synonymous. Generally, the term ‘seafood’ is understood to stand for ´any form of sea life regarded as foods by humans, prominently (but not exclusively) including fish and shellfish´ [100]. Shellfish include various species of molluscs (e.g., bivalve molluscs such as clams, oysters and mussels and cephalopods such as octopus and squid), crustaceans (e.g., shrimp, crabs and lobster) and echinoderms (e.g., sea cucumbers and sea urchins).

In recent years, a considerable number of scientific studies have been dedicated to analysing the microbiological (and sensory) effects of the CAP treatment of fish and ‘seafoods’. In the following, we will restrict ourselves to seafoods of animal origin.

From a nutritional viewpoint, fish species may be conveniently divided into oily fish (i.e., fish in which lipids in the soft tissues and the coelom are present as oil) and whitefish [101]. Fish oil from ‘oily fish’ species is valued for its in vitamin and omega-3-fatty acid contents [102]. Arguably, studies on the antibacterial action of CAP in oily fish species need to consider lipid oxidation as well.

Rathod et al. [103] concluded that CAP treatment would retard bacterial spoilage (i.e., protein degradation and lipid oxidation) and, thus, CAP could be recommended as a minimal processing intervention for preserving the quality of seafood of animal origin.

### 4.1. Oily Fish

#### 4.1.1. Atlantic Mackerel

Atlantic mackerel [(*Scomber scombrus*), a swarm fish caught in coastal waters, is one of the most abundant fish species in Europe, containing high levels of long-chain polyunsaturated fatty acids (PUFAs), which, consequently, are highly susceptible to oxidation, and, thus, may cause the production of off-flavours and -odours. The effects of CAP (generated from ambient air) on fillets of fresh mackerel were investigated in 2017 [104]. When fresh mackerel fillets were stored in packages and subjected to CAP using a ‘large-gap’ (i.e., the target is placed between the electrodes, see Section 2.4) DBD (70–80 kV for 1, 3 and 5 min), microbiological and quality characteristics were improved significantly. The spoilage bacteria (i.e., psychrotrophic aerobic flora, *Pseudomonas* and Lactic acid bacteria) of mackerel fillets were reduced by ca. 1 log cycle through CAP within 24 h of post-CAP treatment. A significant increase in lipid oxidation parameters (i.e., peroxide values, dienes) was observed in CAP-treated samples. The intensity and duration of CAP treatment of mackerel fillets also have a great impact on their microbiological condition. Nevertheless, no changes in pH and colour (with the exception of L* values) were recorded as a result of CAP treatment. These results imply that CAP could be considered as a means of reducing spoilage bacteria and thus extending the shelf life of mackerel, provided an antioxidant is added during storage to keep lipid oxidation in check. Recently, Trevisani et al. [105] confirmed that mackerel subjected to CAP using a DBD (3.8 kV at 12.7 Hz) did not, with the exception of slightly higher lightness (L*) values attributed to the oxidation of haemoproteins [106], appreciably change sensory traits when stored for 5 days at 4 °C. CAP had been generated from ambient air, and exposure was under wet conditions. Trevisani et al. [105] conclude that the treatment of fish fillets before long distance transportation (under challenging environmental conditions) may contribute to safety and extend their shelf life.

#### 4.1.2. Tuna

The latest update on global fish consumption shows that Tuna (*Thunnus obesus*) is the world’s most popular fish food and Pan et al. [107] recently investigated the effects of CAP on its quality. Tuna slices of 10 g (2.5 cm × 5 cm) were subjected to 40 kV CAP, generated from ambient air, in a ´large gap’ DBD design (i.e., the electrodes are at a distance which allows the sample to be placed between them, see also Section 2.4). No changes in sensory effects on tuna sashimi (i.e., raw, fresh, finely filleted tuna) were reported, but significantly different levels of a volatile compound (‘1-hexanol’, a chemical known to indicate the level of protection against flavour changes that negatively affect shelf life) indicate a superior ‘freshness’ of CAP-treated tuna [107].

#### 4.1.3. Herring

Albertos et al. [108] investigated the use of a ‘large gap’ DBD design to generate a CAP discharge within the headspace of packaged herring *(Clupea harengus*) fillets, and its effects on microbiological and quality markers after 11 days storage at 4 °C. DBD plasma treatment conditions were 70 kV or 80 kV for 5 min treatment time. The results showed that the microbial load (total aerobic mesophilic-/total aerobic psychrotrophic bacteria, *Pseudomonas*, lactic acid bacteria and *Enterobacteriaceae*) was significantly (*p* < 0.05) lower in the treated samples, compared with untreated controls. Samples exposed to the lowest applied voltage better retained key quality factors (i.e., lower oxidation and less colour modification). DBD-treatment caused a reduction in ‘trapped water’ in the myofibrillar network, as assessed by the ‘low-field nuclear magnetic resonance of protons‘ technique. The results indicate that in-package DBD plasma treatment could be employed as an effective treatment for reducing spoilage bacteria in highly perishable fish products.

### 4.2. Whitefish

#### 4.2.1. Alaska Pollock

Choi et al. [109] investigated the effect of a corona discharge plasma jet (CDPJ) using ambient air on microbial reduction and the physical-chemical and sensory characteristics of dried Alaska Pollock (*Pollachius pollachius,* a cod species) shreds. All of the spoilage or pathogenic bacteria, moulds and yeasts researched were significantly reduced by 1–2.3 log units. A 3 min exposure reduced the water content from 15 to 8.6%, and a significant increase in Thiobarbituric Acid Reactive Substances (TBARS) was observed. Although individual colour coordinates (L*, a*, b*) remained unchanged, a significant increase in ΔE (up to 2.2 units) was noted. This change, although rated as ‘distinct’ [110], would not necessarily be perceived by consumers. Delta-E [(ΔE = (ΔL*)^2^ + (Δa*)^2^ + (Δb*)^2^)^0.5^] [111] was used as a proxy for visually perceived colour changes. ΔE is a single number that represents the ‘distance’ between two colours, the idea being that a ΔE of 1 is the smallest colour difference the human eye can perceive [112]. More specifically, ΔE < 2 indicates a colour change visible to an experienced observer only and ΔE > 5 indicates the impression of two different colours [111].

Likewise, there was a change in texture, with CAP-exposed samples rated as ‘more crispy’. Other sensory quality parameters were not affected. Both oxidation and drying may have contributed to the changes in ΔE, and crispiness was most likely affected by drying. The authors concluded that 2 min exposure time would yield an optimal condition in terms of quality traits and the inactivation of bacteria.

#### 4.2.2. Hairtail Fish

Koddy et al. [113] studied the effect of CAP at 50 kV with different treatment times on the crude protease extract and muscle protein from Hairtail fish ((*Trichiurus Lepturus*), a saltwater species (‘daiyu’ in Chinese), one of the most popular fish species on the Chinese table). The results suggest that implementing CAP at 50 kV can inhibit the activity of crude protease extract to the lowest value of 0.035 units/mg protein after 240 s. Protein oxidation indices (carbonyls and sulfhydryl) varied significantly after crude protease enzyme was exposed to plasma active species. An enhancement in the colour and water-holding capacity properties was observed in hairtail samples treated with CAP. Therefore, CAP treatment could be used as an effective non-thermal method to maintain the quality of hairtail fish and extend the shelf life. Supplementary research is needed to provide knowledge concerning the effect of CAP treatment on the lipid oxidation of hairtail muscle.

### 4.3. Shrimps

#### 4.3.1. Pacific White Shrimp and Greasyback Shrimp

In recent years, some CAP research has been conducted on Pacific white shrimp ((*Lito-)Penaeus vannamei*), primarily with a view to investigate if ‘melanosis’ (the enzymatic oxidation of shrimps leading to ‘black spots’, a condition associated with serious economic losses) can be counteracted by a 10 min CAP treatment (DBD configuration, plasma generated from air, 500 Hz, 40 kV; [114]). Although, immediately after CAP exposure, microbiological variables (i.e., counts of mesophilic/psychrotrophic bacteria, *Staphylococcus*) were lower in the treated group than in the control group, no significant differences were noted after 3 and 6 days of storage. However, treated samples had significantly lower pH values, higher water-binding capacity and (for a storage period of up to 9 days), a lower cooking loss than controls, and ΔE values were lower. The assessment of overall sensory quality (index composed from six factors) indicated that the shelf life of plasma-treated shrimps was >4 days longer than that of the controls (14.1 vs. 9.8 days), which would increase marketability enormously.

Recently, Elliot et al. [115] suggested to incorporate CAP in the traditional processing chain of fresh shrimp (*Penaeus vannamei*), i.e., immediately after the traditional double wash preceding refrigerated storage at 4 °C for 12 days—a minimal treatment with cold plasma (DBD, 60 kV, 69/90/120 or 150 s). This treatment results in more desirable quality outcomes. The latter are characterised by low malondialdehyde concentration, low volatile nitrogen products content and comparable proximate composition as compared with the traditional approach without CAP. Texture, pH and colour are remarkably retained at 120 and 150 s of CAP pre-treatment and protein degradation is negligible up to 90 s, as opposed to 120 and 150 s of pre-treatment [115].

Whereas most trials subjecting shrimps to CAP rely on direct treatment of the surface with the gases, and positive effects, particularly on some reported physical-chemical effects, there are few data on microbiological effects. Several years ago, a group of Chinese researchers [116] reported the superior quality of Greasyback Shrimps (*Metapenaeus ensis*) that had been stored in ice prepared from plasma-activated water (PAW), as compared with tap water. The former treatment group exhibited less microbial growth, thus extending the storage life 4 to 8 days. During storage, pH values remained < 7.7 and less off-colours and surface ‘hardness’ were observed. The total volatile basic nitrogen (TVBN) values remained at levels < 20 mg/100 g, i.e., at levels significantly lower (*p* < 0.05) than the controls not stored in ice prepared from CAP-treated water.

#### 4.3.2. A Short Note on Freshwater Shrimps’ Role in Spreading Antibiotics Resistance

Recently, serious concerns have been raised about veterinary drug residues in imported shrimp from Asia [117]. The Chinese freshwater grass-shrimp (*Paleomonetes sinensis,* generally used as an aquarium ‘cleaner’ rather than as food for human consumption) is a shrimp species that eats dead/decaying plants and animals). It has recently been indicated that this shrimp could play a significant role in spreading antibiotic resistance.

In the USA, laboratory tests have shown that most frozen freshwater shrimp samples imported from Asia (e.g., Thailand, China, India, Indonesia, Bangladesh) and Ecuador contain residues of, e.g., oxytetracyclin, nitrofurantoin, fluoroquinolone and malachite green, i.e., antibiotics that are restricted or banned under US food standards. Apparently, existing screening protocols and enforcement measures are insufficient to prevent this from happening. There are also serious doubts if, currently, adequate labelling rules are followed [117].

The use of freshwater shrimp as a human food is generally advised against [118]. Although freshwater shrimp is entirely edible, its reputation is that it is ‘not worth the effort’ (as there is hardly any meat to eat, and it is classified as a ‘gooey’ (syrupy, viscous, sticky) substance that is better left for ‘monster fishes’ to eat) [118,119].

Arguably, alternative antibacterial interventions could help to reduce the use of antimicrobials in shrimp production. This might include the application of CAP to sanitise the water in the breeding/holding pens.

### 4.4. Squid

Choi et al. [120,121] investigated the effect of a CDPJ, with plasma generated from air, on microbial reduction and the physical-chemical and sensory characteristics of semi-dried and dried squid *(Todarodes pacificus)* shreds. All the spoilage or pathogenic bacteria, moulds and yeasts researched were significantly reduced. In semi-dried squid shreds, exposure times > 3 min resulted in a change of flavour and significant increase in TBARS and L*and b* values, with ΔE up to 6.6 after 10 min exposure. The levels of TVBN and trimethylamine were not affected. The overall sensory acceptance was not impaired. Likewise, a 3 min. exposure of dried shreds resulted in increases in L* (ΔE maximum 2.5) and TBARS, indicative for oxidative changes. Again, the overall acceptance was not impaired. The authors concluded that a 2 min. exposure would warrant a sufficient reduction in bacteria without compromising product characteristics.

### 4.5. Molluscs (Mussels and Oysters)

Mussels and oysters have been implicated in foodborne poisoning, either by contaminant bacteria or viruses [122,123,124,125,126,127,128,129,130,131]. Unlike fish, this type of seafood is often traded alive and some species are even consumed raw (e.g., oysters). Thus, no traditional food processing techniques with antimicrobial or antiviral effect (e.g., heat treatment; [132]) can be applied. Choi et al. [133] exposed an oyster (*Crassostrea gigas*) slurry to plasma generated by a jet-type CAP device for 30 min and could demonstrate a reduction (>1 log) in human norovirus without compromising the colour and pH of the oyster [133]. Although the authors selected a highly relevant viral pathogen, biosafety concerns make the use of surrogate viruses more feasible, which is an issue discussed in more detail in the following section.

Csadek et al. [134] studied the inactivation of surrogate viruses on an oyster slurry, but employed a DBD instead of a jet-type plasma generator. Plasma was generated from ambient air. The authors observed a higher antiviral effect towards a double-stranded DNA virus (Equid Alphaherpesvirus 1, EHV-1; 2.3–2.8 log) than against a single-stranded RNA virus (Bovine Coronavirus, BCoV; 1.4–1.0 log) in Dulbecco’s Modified Eagle’s Medium (DMEM). Plasma generated at low power (ozone dominated) had a higher virus inactivation effect than was the case at high power (nitrogen dioxide dominated). Plasma exposure caused a decline of glucose contents in DMEM, which might have been caused by a reaction of carbohydrates with amino acids (Maillard reaction). The exposure of an oyster slurry to CAP did not result in any change of pH and colour, corroborating the findings of Choi et al. [133]. However, the oyster matrix resembles a buffered medium, and, thus, pH changes were not really expected. Regarding colour, different mechanisms may apply than in muscle foods, since the electron acceptor is haemocyanin instead of haemoglobin [135]. Thus, the absence of colour changes is maybe less suitable for assessing CAP-induced changes. The authors observed an accumulation of nitrogen in the oyster slurry due to CAP exposure, which could only in part be explained by higher nitrate and nitrite contents. The (entirely plausible) assumption that nitrate from the plasma reacted with the compounds of the oyster matrix remains to be substantiated.

Both Choi et al. [133] and Csadek et al. [134] studied oyster tissue and not live oyster, and it was a contamination scenario, not an infection. However, the antiviral effects of CAP exposure can also be expected for live oysters, whereas the significance of the observed nitrogen accumulation in oyster slurry for live animals is not entirely clear. It can be assumed that the exposure of the cells to nitrate is a stressor for cell homoeostasis. Studies on exposure to ACP of oyster cell monolayers with or without viral contaminants might allow the obtainment of an estimate if the extent and the benefit of antiviral action are counteracted by oyster cell damage.

Admittedly, the direct exposure of oysters to CAP is not very practical, compared to sanitising the (salt) water in the holding tanks. Although the concept of applying CAP to reduce viral contamination in water is promising [136], it will largely depend on the composition of the plasma species. When NO_x_ are generated, they will be dissolved in water and lower the pH [134]. This pH drop is an additional stressor for the oysters.

**Figure 2:**
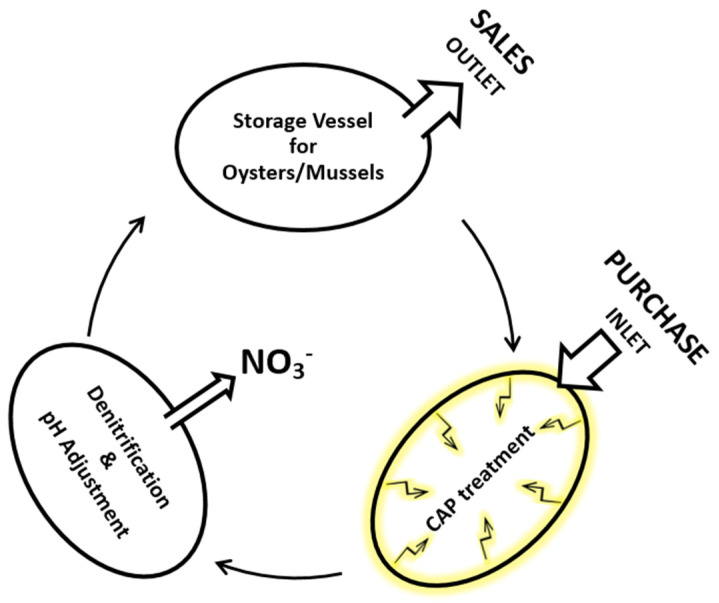
Concept of a circulation system for denitrification and cold atmospheric plasma treatment of stored molluscs (source: authors).

A CAP-based system for sanitising water in holding tanks for live oysters could allow the water to circulate from the holding tank via a compartment for CAP exposure to a station adjusting the pH (either by adding alkali or by denitrification) and again back to the holding tank. Denitrification is usually a biological process, but there are also chemical systems available [137,138]. Based on these references, the authors of this article suggest studies on a circulating system (Figure 2), in which CAP treatment would prevent re-infections or new infections and eventually reduce the pathogen load of already-infected oysters and mussels.

## 5. A Note on Food-Contaminating Viruses; Why Surrogate Viruses Are Used for Studying the Virucidal Effect of Cold Atmospheric Plasma

While food-transmitted pathogenic viruses are of concern for food safety, food security is threatened by viruses causing disease in production animals, e.g., Newcastle disease virus (NDV) and porcine reproductive and respiratory syndrome virus (PRRSV), sometimes with zoonotic potential (highly pathogenic avian influenza virus, HPAIV). It can be assumed that CAP would effectuate virus inactivation on the surface of biological materials the same way as it acts on food surfaces.

The most recent authoritative review on cold plasma effects on viruses stems from Filipić et al. [139], who acknowledge that, so far, insufficient data are available that would allow selecting the correct treatment options. Unfortunately, the literature on plasma effects against foodborne viruses is rather scarce and relevant parameters (including a treatment duration that would allow optimal interaction with contaminated material) have not yet been studied sufficiently. The biosecurity problems associated with studying pathogenic viruses have been mentioned above and might also be a reason why so few studies have been conducted on viruses. Thus, it is worth addressing the use of surrogate viruses in more detail.

The literature on foodborne viruses focuses on noroviruses, enteric adenoviruses and hepatitis A virus, which are the leading causes of acute gastroenteritis, the second most infectious disease worldwide, responsible for high levels of hospitalisation and mortality [139,140]. The infectivity of these agents explains why research on the effects of CAP on these viruses is rather limited and usually relies on the use of ‘surrogate’ viruses that are relatively easy to culture/propagate and are safe to work with [141]. By the same token, it has been proposed to use bacteriophages as surrogates or indicators for the presence of enteric infectious viruses in wastewater [140].

Pathogenic viruses have always posed a great risk to humans and animals alike, and pandemics can quickly wreak havoc on the livelihood of millions of people, as currently seen due to SARS-CoV-2. Apart from human-to-human transmission, contaminated surfaces are another source of viral infections, and the ingestion of dangerous pathogens on food surfaces frequently results in significant morbidities and mortalities [142]. Apart from considerable evidence of the microbicidal effect of atmospheric-pressure cold plasma on bacteria and fungi, several studies indicate that CAP treatment also induces virus inactivation and is therefore considered a promising tool to combat human pathogenic viruses [143].

An evaluation of the efficacy of inactivation methods for pathogenic human viruses may imply significant health hazards, which may require specialised buildings and equipment, such as biosafety level 3 (BSL-3) laboratories, which are not generally available. Furthermore, such resources are not only expensive, but also depend on specially trained personnel; thus, extensive testing of such viruses is often not economically justified. As a consequence, surrogate viruses that can be handled in BSL-1 or BSL-2 facilities are usually favoured for testing and optimising plasma technology [142]. In addition, most food-related viruses cannot be propagated in cell culture (e.g., hepatitis viruses and caliciviruses), and/or do not cause cytopathic effects, which would be required to directly assess a viral reduction caused by, e.g., cold plasma in cell culture. Thus, ways to inactivate infectious viruses are most commonly investigated by the use of cultivable surrogate viruses, or by the detection of viral nucleic acids by real-time polymerase chain reaction (RT-)PCR, which, however, does not provide information about infectivity [141], unless free DNA or RNA molecules released from destroyed viral particles are removed prior to PCR by DNase or RNase treatment, respectively [144].

Surrogate viruses should be closely related or have very similar traits to mimic the pathogen of interest as well as possible, including biological, biophysical and biochemical characteristics [141]. For instance, noroviruses are single-stranded, non-enveloped RNA viruses belonging to the family *Caliciviridae*. Thus, viruses from the same family are the best surrogate choice, such as feline calicivirus, which is cultivable (but does not cause a cytopathic effect) and has been used as a surrogate for norovirus in several studies since the 1970s [141]. When murine norovirus, which is even more closely related to food-contaminating noroviruses, was discovered in 2003, researchers turned to this virus, as it is also resistant to low pH values. However, when it was found that the murine norovirus is highly sensitive to alcohols, the search expanded to other cultivable caliciviruses [141], which shows that, aside from genetic and morphologic similarity, there are several other features of a surrogate that have to be considered.

## 6. Conclusions and Future Perspectives

Cold plasma technology is a cornerstone of modern society given its ubiquitous use in materials manufacturing applications (e.g., semiconductor fabrication and polymer treatment). Recently, its action on biological material has been studied extensively, with three major fields of application, i.e., medical treatments (e.g., cell regeneration and wound healing), non-thermal surface disinfection and food science. Food science applications are more complex, since they aim at inactivating contaminant bacteria and viruses on food surfaces, but need to take into account that plasma species will also react with the food matrix.

In this contribution, we have shown that cold atmospheric-pressure plasma can be generated with low energy consumption simply using air as the precursor for the generation of reactive chemical species. A large number of studies have shown that not only the gas composition, but also the mode of plasma generation and the spatial-temporal distance from the plasma source to the target govern which chemical species will reach the surface of the target, which in turn affects the ability of the CAP to inactivate microorganisms. In this contribution, we have shown that, on the surfaces of meat and seafood treated with indirect CAP systems, ozone and nitrate are the major reactive gas species, and their inactivating action on bacteria and viruses is known. By the same token, their effects on the food matrix are well described (i.e., lipid oxidation, the action of NO on haem pigments and eventually the oxidation of (haem) proteins, oyster tissue) but are not plasma-specific.

In summary, this contribution has shown that atmospheric-pressure cold plasma is capable of effectively reducing the load of bacteria and viruses on the surfaces of fresh as well as processed meat and seafood. Available data suggest, in most cases, no or only negligible effects of plasma species on food matrices in terms of chemical composition, colour and physical-chemical properties; nitrogen accumulation on oyster tissue being an exception.

Going forward, the insight gained from an expert opinion published in 2012 must be considered, where the diversity of plasma-generation devices, conditions and treatment protocols was identified as a drawback in conducting a detailed risk assessment with respect to the applicability of CAP in sanitising fresh meat and thus could not clearly answer if such treated food items would be ‘novel foods’ according to EU legislation [55]. Given the multitude of data generated in the last 10 years, the application of atmospheric-pressure cold plasma on meat and fish (products) deserves a formal re-assessment in this respect.

## Data Availability

Not applicable.

## References

[1] Lipinski B. (2020). Why do animal-based food loss and waste matter?. Anim. Front..

[2] FAO. Food and Agriculture Organization of the United Nations Food Wastage Footprint and Climate Change, Rome Italy, 2015. https://www.fao.org/3/bb144e/bb144e.pdf.

[3] Warner R.D., Greenwood P.L., Pethick D.W., Ferguson D.M. (2010). Genetic and environmental effects on meat quality. Meat Sci..

[4] Jan A.T., Lee E.J., Ahmad S., Choi I. (2016). Meeting the meat: Delineating the molecular machinery of muscle development. J. Anim. Sci. Technol..

[5] Mohammadabadi M., Bordbar F., Jensen J., Du M., Guo W. (2021). Key genes regulating skeletal muscle development and growth in farm animals. Animals.

[6] Mohammadi A., Nassiry M.R., Mosafer J., Mohammadabadi M.R. (2009). Distribution of BoLA-DRB3 allelic frequencies and identification of a new allele in the Iranian cattle breed Sistani (*Bos indicus*). Russ. J. Genet..

[7] Mossel D.A.A. (1989). Adequate protection of the public against food-transmitted diseases of microbial aetiology. Achievements and challenges half a century after the introduction of the Prescott-Meyer-Wilson strategy of active intervention. Int. J. Food Microbiol..

[8] FAO. Food and Agriculture Organization of the United Nations (1996). Rome Declaration on World Food Security. World Food Summit, 13–17 November 1996, Rome, Italy. https://www.fao.org/3/w3613e/w3613e00.htm.

[9] UN World Commission on Environment and Development (1987). Report of the World Commission on Environment and Development: Our Common Future. https://sustainabledevelopment.un.org/content/documents/5987our-common-future.pdf.

[10] European Commission Farm to Fork Strategy; for a Fair, Healthy and Environmentally-Friendly System. https://food.ec.europa.eu.

[11] Warne G.R., Williams P.M., Pho H.Q., Tran N.N., Hessel V., Fisk I.D. (2021). Impact of cold plasma on the biomolecules and organoleptic properties of foods: A review. Food Sci..

[12] Jadhav H.B., Annapure U. (2021). Consequences of non-thermal cold plasma treatment on meat and dairy lipids—A review. Future Foods.

[13] Olatunde O.O., Singh A., Shiekh K.A., Nuthong P., Benjakul S. (2021). Effect of High Voltage Cold Plasma on Oxidation, Physiochemical, and Gelling Properties of Myofibrillar Protein Isolate from Asian Sea Bass (*Lates calcarifer*). Foods.

[14] Domínguez R., Pateiro M., Munekata P.E.S., Zhang W., Garcia-Oliveira P., Carpena M., Prieto M.A., Bohrer B., Lorenzo J.M. (2022). Protein Oxidation in Muscle Foods: A Comprehensive Review. Antioxidants.

[15] Nawaz A., Irshad S., Ali Khan I., Khalifa I., Walayat N., Aadil R.M., Kumar M., Wang M., Chen F., Cheng K.-W. (2022). Protein oxidation in muscle-based products: Effects on physicochemical properties, quality concerns, and challenges to food industry. Food Res. Int..

[16] Raizer Y.P. (1997). Gas Discharge Physics.

[17] Lieberman M.A., Lichtenberg A.J. (2005). Principles of Plasma Discharges and Materials Processing.

[18] Bourke P., Ziuzina D., Han L., Cullen P.J., Gilmore B.F. (2017). Microbiological Interactions with Cold Plasma. J. Appl. Microbiol..

[19] Martines E. (2020). 2020. Interaction of Cold Atmospheric Plasmas with Cell Membranes in Plasma Medicine Studies. Jpn. J. Appl. Phys..

[20] Mendis D.A., Rosenberg M., Azam F. (2000). A Note on the Possible Electrostatic Disruption of Bacteria. IEEE Trans. Plasma Sci..

[21] Lackmann J.-W., Schneider S., Edengeiser E., Jarzina F., Brinckmann S., Steinborn E., Havenith M., Benedikt J., Bandow J.E. (2013). Photons and Particles Emitted from Cold Atmospheric-Pressure Plasma Inactivate Bacteria and Biomolecules Independently and Synergistically. J. R. Soc. Interface..

[22] Bruggeman P.J., Iza F., Brandenburg R. (2017). Foundations of Atmospheric Pressure Non-Equilibrium Plasmas. Plasma Sources Sci. Technol..

[23] Brandenburg R. (2017). Dielectric Barrier Discharges: Progress on Plasma Sources and on the Understanding of Regimes and Single Filaments. Plasma Sources Sci. Technol..

[24] Laroussi M. (2002). Nonthermal Decontamination of Biological Media by Atmospheric-Pressure Plasmas: Review, Analysis, and Prospects. IEEE Trans. Plasma Sci..

[25] Laroussi M., Bekeschus S., Bogaerts A., Fridman A., Lu X., Miller V., Laux C., Walsh J., Jiang C., Liu D. (2022). Low-Temperature Plasma for Biology, Hygiene, and Medicine: Perspective and Roadmap. IEEE Trans. Radiat. Plasma Med. Sci..

[26] Brun P., Vono M., Venier P., Tarricone E., Deligianni V., Martines E., Zuin M., Spagnolo S., Cavazzana R., Cardin R. (2012). Disinfection of Ocular Cells and Tissues by Atmospheric-Pressure Cold Plasma. PLoS ONE.

[27] Diwan R., Debta F.M., Deoghare A., Ghom S., Khandelwhal A., Sikdar S., Ghom A. (2011). Plasma therapy; An overview. J. Indian Acad. Oral Med. Radiol..

[28] Lackmann J.-W., Bandow J.E. (2014). Inactivation of microbes and macromolecules by atmospheric-pressure plasma jets. Appl. Microbiol. Biotechnol..

[29] Zimmermann J. Cold Atmospheric Plasma in Medicine—From Basic Research to Application, Max Planck Institute of Pathology, Technical University Munich, Habilitation Treatise; 2013. http://mediatum.ub.tum.de/?id=1219297.

[30] Bekeschus S., von Woedtke T., Emmert S., Schmidt A. (2021). Medical Gas Plasma-Stimulated Wound Healing: Evidence and Mechanisms. Redox Biol..

[31] Melotti L., Martinello T., Perazzi A., Martines E., Zuin M., Modenese D., Cordaro L., Ferro S., Maccatrozzo L., Iacopetti I. (2021). Could cold plasma act synergistically with allogeneic mesenchymal stem cells to improve wound skin regeneration in a large size animal model?. Res. Vet. Sci..

[32] Scholtz V., Pazlarova J., Souskova H., Julak J. (2015). Nonthermal Plasma—A Tool for Decontamination and Disinfection. Biotechnol. Adv..

[33] Misra N.N., Schlüter O., Cullen P.J., Misra N.N., Schlüter O., Cullen P.J. (2016). Chapter 1—Plasma in Food and Agriculture. Cold Plasma in Food and Agriculture.

[34] Csadek I., Paulsen P., Bak K.H., Smulders F.J.M. (2021). Application of atmospheric pressure cold plasma (ACP) on meat and meat products. Part 1. Effects on bacterial surface flora. Fleischwirtschaft.

[35] Shimizu T., Sakiyama Y., Graves D., Zimmerman J., Morfill G. (2012). The dynamics of ozone generation and mode transition in air surface micro-discharge plasma at atmospheric pressure. New J. Phys..

[36] Reuter S., Winter J., Iseni S., Schmidt-Bleker A., Dünnbier M., Masur K., Wende K., Weltmann K.-D. (2015). The Influence of Feed Gas Humidity Versus Ambient Humidity on Atmospheric Pressure Plasma Jet-Effluent Chemistry and Skin Cell Viability. IEEE Trans. Plasma Sci..

[37] Lis K.A., Boulaaba A., Binder S., Li Y., Kehrenberg C., Zimmermann J.L., Klein G., Ahlfeld B. (2018). Inactivation of *Salmonella* Typhimurium and *Listeria monocytogenes* on Ham with Nonthermal Atmospheric Pressure Plasma. PLoS ONE.

[38] Katsigiannis A.S., Bayliss D.L., Walsh J.L. (2022). Cold Plasma for the Disinfection of Industrial Food-contact Surfaces: An Overview of Current Status and Opportunities. Comp. Rev. Food Sci. Food Saf..

[39] López M., Calvo T., Prieto M., Múgica-Vidal R., Muro-Fraguas I., Alba-Elías F., Alvarez-Ordóñez A. (2019). A Review on Non-Thermal Atmospheric Plasma for Food Preservation: Mode of Action, Determinants of Effectiveness, and Applications. Front. Microbiol..

[40] Hasan M.I., Walsh J.L. (2017). Influence of Gas Flow Velocity on the Transport of Chemical Species in an Atmospheric Pressure Air Plasma Discharge. Appl. Phys. Lett..

[41] Misra N.N., Yepez X., Xu L., Keener K. (2019). In-Package Cold Plasma Technologies. J. Food Eng..

[42] Ziuzina D., Patil S., Cullen P.J., Keener K.M., Bourke P. (2013). Atmospheric Cold Plasma Inactivation of *Escherichia coli* in Liquid Media inside a Sealed Package. J. Appl. Microbiol..

[43] Huang M., Wang J., Zhuang H., Yan W., Zhao J., Zhang J. (2019). Effect of In-Package High Voltage Dielectric Barrier Discharge on Microbiological, Color and Oxidation Properties of Pork in Modified Atmosphere Packaging during Storage. Meat Sci..

[44] Patange A., Boehm D., Bueno-Ferrer C., Cullen P.J., Bourke P. (2017). Controlling *Brochothrix thermosphacta* as a Spoilage Risk Using In-Package Atmospheric Cold Plasma. Food Microbiol..

[45] Wang J., Zhuang H., Hinton A., Zhang J. (2016). Influence of in-package cold plasma treatment on microbiological shelf life and appearance of fresh chicken breast fillets. Food Microbiol..

[46] Levif P., Séguin J., Moisan M., Soum-Glaude A., Barbeau J. (2011). Packaging materials for plasma sterilization with the flowing afterglow of an N_2_—O_2_ discharge: Damage assessment and inactivation efficiency of enclosed bacterial spores. J. Phys. D Appl. Phys..

[47] Brayfield R.S., Jassem A., Lauria M.V., Fairbanks A.J., Keener K.M., Garner A.L. (2018). Characterization of High Voltage Cold Atmospheric Plasma Generation in Sealed Packages as a Function of Container Material and Fill Gas. Plasma Chem. Plasma Process.

[48] Bauer A., Ni Y., Bauer S., Paulsen P., Modic M., Walsh J.L., Smulders F.J.M. (2017). The effects of atmospheric pressure cold plasma treatment on microbiological, physical-chemical and sensory characteristics of vacuum packaged beef loin. Meat Sci..

[49] Rød S.K., Hansen F., Leipold F., Knøchel S. (2012). Cold atmospheric pressure plasma treatment of ready-to-eat meat: Inactivation of *Listeria innocua* and changes in product quality. Food Microbiol..

[50] Roh S., Oh Y., Le S., Kann J., Min S. (2020). Inactivation of *Escherichia coli* O157:H7, *Salmonella*, *Listeria monocytogenes* and Tulane virus in processed chicken breast via atmospheric in-package cold plasma treatment. LWT Food Sci. Technol..

[51] Jayasena D., Kim H., Yong H., Park S., Kim K., Choe W., Jo C. (2015). Flexible thin-layer dielectric barrier discharge plasma treatment of pork butt and beef loin: Effects on pathogen inactivation and meat-quality attributes. Food Microbiol..

[52] Harrison W.A., Griffith C.J., Tennant D., Peters A.C. (2001). Incidence of *Campylobacter* and *Salmonella* isolated from retail chicken and associated packaging in South Wales. Lett. Appl. Microbiol..

[53] (2015). Regulation (EU) 2015/2283 of the European Parliament and of the Council of 25 November 2015 on novel foods, amending Regulation (EU) No 1169/2011 of the European Parliament and of the Council and repealing Regulation (EC) No 258/97 of the European Parliament and of the Council and Commission Regulation (EC) No 1852/2001. Off. J. Eur. Union.

[54] Cooperation in Science and Technology (COST 013/20) Decision 24 March; Memorandum of Understanding for the Implementation of the COST Action “Plasma Application for Smart and Sustainable Agriculture (P/Agri) CA 19110”, Brussels, 16 pp., COST, Avenue Boulevard 21, Brussels, Belgium, 2020. https://plagri.eu/wp-content/uploads/2021/02/CA19110-e.pdf.

[55] 55. German Research Community (Deutsche Forschungsgemeinschaft). Stellungnahme Zum Einsatz von Plasmaverfahren zur Behandlung von Lebensmitteln [Opinion on the Application of Plasma-technology for Treatment of Foods], 2012. www.dfg.de/sklm.

[56] Ekezie C., Suan D., Cheng J. (2017). Review on recent advances in cold plasma technology for the food industry: Current applications and future trends. Trends Food Sci. Technol..

[57] Nørrung B., Buncic S. (2008). Microbial safety of meat in the European Union. Meat Sci..

[58] Das A.K., Nanda P.K., Das A., Biswas S., Singh R.L., Mondal S. (2019). Hazards and Safety Issues of Meat and Meat Products. Food Safety and Human Health.

[59] (2002). Regulation (EC) No 178/2002 of the European Parliament and of the Council of 28 January 2002 laying down the general principles and requirements of food law, establishing the European Food Safety Authority and laying down procedures in matters of food safety. Off. J. Eur. Union.

[60] Pearce R.A., Bolton D.J., Sheridan J.J., McDowell D.A., Blair I.S., Harrington D. (2004). Studies to determine the critical control points in pork slaughter hazard analysis and critical control point systems. Int. J. Microbiol..

[61] Antic D., Houf K., Michalopoulou E., Blagojevic B. (2021). Beef abattoir interventions in a risk-based meat safety assurance system. Meat Sci..

[62] Paulsen P., Smulders F.J.M., Zeuthen P., Bogh-Sorensen L. (2003). Combining natural antimicrobial systems with other preservation techniques: The case of meat. Food Preservation Techniques.

[63] Aymerich T., Picouet T.A., Monfort J.M. (2008). Decontamination technologies for meat products. Meat Sci..

[64] Loretz M., Stephan R., Zweifel C. (2011). Antibacterial activity of decontamination treatments for cattle hides and beef carcasses. Food Control.

[65] Buncic S., Sofos J. (2012). Interventions to Control *Salmonella* Contamination during Poultry, Cattle and Pig Slaughter. Food Res. Int..

[66] Buncic S., Nychas G.-J., Lee M.R.F., Koutsoumanis K., Hébraud M., Desvaux M., Chorianopoulos N., Bolton D., Blagojevic B., Antic D. (2014). Microbial pathogen control in the beef chain: Recent research advances. Meat Sci..

[67] Albert T., Braun P.G., Saffaf J., Wiacek C. (2021). Physical Methods for the Decontamination of Meat Surfaces. Curr. Clin. Microbiol. Rep..

[68] Zdolec N., Kotsiri A., Houf K., Alvarez-Ordóñez A., Blagojevic B., Karabasil N., Salines M., Antic D. (2022). Systematic Review and Meta-Analysis of the Efficacy of Interventions Applied during Primary Processing to Reduce Microbial Contamination on Pig Carcasses. Foods.

[69] Gill C.O., Bedard D., Jones T. (1997). The decontaminating performance of a commercial apparatus for pasteurizing polished pig carcasses. Food Microbiol..

[70] Gill C.O., Jones T., Badoni M. (1998). The effects of hot water pasteurizing treatments on the microbiological conditions and appearances of pig and sheep carcasses. Food Res. Int..

[71] Alban L., Sørensen L.L. (2010). Hot-water decontamination is an effective way of reducing risk of *Salmonella* in pork. Fleischwirtschaft.

[72] Paulsen P., Smulders F.J.M., Smulders F.J.M., Collins J.D. (2004). Reduction of the microbial contamination of carcasses and meat cuts with particular reference to the application of organic acids. Food Safety and Veterinary Public Health, Volume 2: Safety Assurance during Food Processing.

[73] Smulders F.J.M., Wellm G., Hiesberger J., Rohrbacher I., Bauer A., Paulsen P. (2011). Microbiological and sensory effects of the combined application of hot-cold organic acid sprays and steam condensation at subatmospheric pressure for decontamination of inoculated pig tissue surfaces. J. Food Prot..

[74] Smulders F.J.M., Wellm G., Hiesberger J., Bauer A., Paulsen P. (2012). The potential of the combined application of hot water sprays and steam condensation at subatmospheric pressure for decontaminating inoculated pig skin and muscle surfaces. Food Control.

[75] EFSA Panel on Biological Hazards (BIOHAZ) (2011). Scientific Opinion on the evaluation of the safety and efficacy of lactic acid for the removal of microbial surface contamination of beef carcasses, cuts and trimmings. EFSA J..

[76] Silano V., Barat Baviera J.M., Bolognesi C., Brüschweiler B.J., Chesson A., Cocconcelli P.S., Crebelli R., Gott D.M., Grob K., EFSA Panel on Food Contact Materials, Enzymes and Processing Aids (CEP) (2018). Evaluation of the safety and efficacy of the organic acids lactic and acetic acids to reduce microbiological surface contamination on pork carcasses and pork cuts. EFSA J..

[77] (2013). Commission Regulation (EU) No 101/2013 of 4 February 2013 concerning the use of lactic acid to reduce microbiological surface contamination on bovine carcasses. Off. J. Eur. Union.

[78] Choi S., Puligundla P., Mok C. (2016). Corona discharge plasma jet for inactivation of *Escherichia coli* O157:H157 and *Listeria monocytogenes* on inoculated pork and its impact on meat quality attributes. Ann. Microbiol..

[79] Yong J., Kim H., Park S., Choe W., Oh M., Jo C. (2014). Evaluation of the Treatment of Both Sides of Raw Chicken Breasts with an Atmospheric Pressure Plasma Jet for the Inactivation of *Escherichia Coli*. Foodborne Pathog. Dis..

[80] Misra N.N., Jo C. (2017). Applications of cold plasma technology for microbiological safety in meat industry. Trends Food Sci. Tech..

[81] Lawrie R.A., Ledward D.A. (2006). Lawrie’s Meat Science.

[82] Pérez-Andrés J.M., Cropotova J., Harrison S.M., Brunton N.P., Cullen P.J., Rustad T., Tiwari B.K. (2020). Effect of Cold Plasma on Meat Cholesterol and Lipid Oxidation. Foods.

[83] Bak K.H., Csadek I., Paulsen P., Smulders F.J.M. (2021). Application of atmospheric pressure cold plasma (ACP) on meat and meat products. Part 2. Effects on the sensory quality with special focus on meat colour and lipid oxidation. Fleischwirtschaft.

[84] Kim J.-S., Lee E.-J., Choi E.H., Kim Y.-J. (2014). Inactivation of *Staphylococcus aureus* on the beef jerky by radio-frequency atmospheric pressure plasma discharge treatment. Innov. Food Sci. Emerg. Technol..

[85] Yong H.I., Lee H., Park S., Park J., Choe W., Jung S., Jo C. (2017). Flexible thin-layer plasma inactivation of bacteria and mold survival in beef jerky packaging and its effects on the meat’s physicochemical properties. Meat Sci..

[86] Yong H.I., Lee S.H., Kim S.Y., Park S., Park J., Choe W., Jo C. (2019). Color development, physiochemical properties, and microbiological safety of pork jerky processed with atmospheric pressure plasma. Innov. Food Sci. Emerg. Technol..

[87] Jung S., Kim H.J., Park S., Yong H.I., Choe J.H., Jeon H.-J., Choe W., Jo C. (2015). The use of atmospheric pressure plasma-treated water as a source of nitrite for emulsion-type sausage. Meat Sci..

[88] Jung S., Kim H.J., Park S., Yong H.I., Choe J.H., Jeon H.J., Choe W., Jo C. (2015). Color Developing Capacity of Plasma-treated Water as a Source of Nitrite for Meat Curing. Korean J. Food Sci. Anim. Resour..

[89] Lee J., Jo K., Lim Y., Jeon H.J., Choe J.H., Jo C., Jung S. (2018). The use of atmospheric pressure plasma as a curing process for canned ground ham. Food Chem..

[90] Yong H.I., Park J., Kim H.-J., Jung S., Park S., Lee H.J., Choe W., Jo C. (2018). An innovative curing process with plasma-treated water for production of loin ham and for its quality and safety. Plasma Process. Polym..

[91] Jo K., Lee S., Yong H.I., Choi Y.-S., Jung S. (2020). Nitrite sources for cured meat products. LWT-Food Sci. Technol..

[92] Alahakoon A.U., Jayasena D.D., Ramachandra S., Jo C. (2015). Alternatives to nitrite in processed meat: Up to date. Trends Food Sci. Technol..

[93] Guerrero-Legarreta I., Batt C.A., Tortorello M.L. (2014). Meat and poultry—Spoilage of Cooked Meat and Meat Products. Encyclopedia of Food Microbiology.

[94] Ganan M., Hierro E., Hospital X.F., Barroso E., Fernández M. (2013). Use of pulsed light to increase the safety of ready-to-eat cured meat products. Food Control.

[95] Possas A., Valdramidis V., García-Gimeno R.M., Pérez-Rodríguez F. (2019). High hydrostatic pressure processing of sliced fermented sausages: A quantitative exposure assessment for *Listeria monocytogenes*. Innov. Food Sci. Emerg. Technol..

[96] Hadjicharalambous C., Grispoldi L., Goga B.C. (2019). Quantitative risk assessment of *Listeria monocytogenes* in a traditional RTE product. EFSA J..

[97] European Commission (2005). Commission Regulation (EC) No 2073/2005 of 15 November 2005 on microbiological criteria for foodstuffs. Off. J. Eur. Union.

[98] Awaiwanont N., Smulders F.J.M., Paulsen P. (2017). Growth potential of *Listeria monocytogenes* in traditional Austrian cooked-cured meat products. Food Control.

[99] Csadek I., Vankat U., Schrei J., Graf M., Bauer S., Pilz B., Schwaiger K., Smulders F.J.M., Paulsen P. Treatment of Ready-to-Eat Cooked Meat Products with Cold Atmospheric Plasma to Inactivate *Listeria* and *Escherichia coli*. Foods.

[100] Wikipedia Seafood. https://en.wikipedia.org/wiki/Seafood.

[101] FSA (Food Standards Agency) What Is an Oily Fish?. https://webarchive.nationalarchives.gov.uk/ukgwa/20101210005807/http://www.food.gov.uk/news/newsarchive/2004/jun/oilyfishdefinition.

[102] NHS (National Health Services) (2018). https://www.nhs.uk/live-well/eat-well/food-types/fish-and-shellfish-nutrition.

[103] Rathod N.B., Chudaman R., Bhagwat P.K., Ozugul F., Benjakul S., Sottawat B., Pilai S., Annapure U.S. (2021). Cold Plasma for the preservation of aquatic food products: An overview. Compr. Rev. Food Sci. Food Saf..

[104] Albertos I., Martin Diana A.B., Cullen P.J., Tiwari B.K., Ojha S.K., Bourke P., Alvarez C., Rico D. (2017). Effects of dielectric barrier discharge (DBD) generated plasma on microbial reduction and quality parameters of fresh mackerel (*Scomber scombrus*) fillets. Innov. Food Sci. Emerg. Technol..

[105] Trevisani M., Cavoli C., Ragni L., Cecchini M., Berardinelli A. (2021). Effect of non-thermal atmospheric plasma on viability and histamine-producing activity of psychrotrophic bacteria in mackerel fillets. Front. Microbiol..

[106] Kulawik P., Kumar Tiwari B. (2018). Recent advancements in the application of non-thermal plasma technology for the seafood industry. Crit. Rev. Food Sci. Nutr..

[107] Pan W., Benjakul S., Sanmartin C., Guidi A., Ying X., Ma L., Weng X., Yu J., Deng S. (2022). Characterization of the Flavor Profile of Bigeye Tuna Slices Treated by Cold Plasma Using E-Nose and GC-IMS. Fishes.

[108] Albertos I., Martin Diana A.B., Cullen P.J., Tiwari B.K., Ojha S.K., Bourke P., Rico D. (2019). Shelf life extension of herring (*Clupea harengus*) using in-package atmospheric plasma technology. Innov. Food Sci. Emerg. Technol..

[109] Choi S., Puligundla P., Mok C. (2016). Microbial decontamination of dried Alaska pollock shreds using corona discharge plasma jet: Effects on physico-chemical and sensory characteristics. J. Food Sci..

[110] Tiwari B.K., Muthukumarappan K., O’Donnell C.P., Cullen P.J. (2008). Effects of sonication on the kinetics of orange juice quality parameters. J. Agric. Food Chem..

[111] CIE (Commission Internationale de L’Éclairage (International Commission on Illumination) (1978). Recommendations on Uniform Color Spaces, Color-Difference Equations, Psychometric Color Terms.

[112] Upton S. Delta E: The Color Difference. Delta E, CHROMIX Colornews, 17. http://www.chromix.com/ColorNews/.

[113] Koddy J.K., Miao W., Hatab S., Tang L., Xu H., Nyaisaba B.M., Chen M., Deng S. (2020). Understanding the role of atmospheric cold plasma (CAP) in maintaining the quality of hairtail (*Trichiurus lepturus*). Food Chem..

[114] de Souza Silva D.A., da Silva Campelo M.C., de Oliviera Soares Rebouças L., de Oliveira Vitoriano J., Alves C., Alves da Silva J.B., de Oliveira Lima P. (2019). Use of cold atmospheric plasma to preserve the quality of white shrimp (*Litopenaeus vannamei*). J. Food Prot..

[115] Elliot M., Chen J., Chen D.-Z., Ekaterina N., Deng S.G. (2022). Effect of cold plasma-assisted shrimp processing chain in biochemical and sensory quality alterations in Pacific White Shrimps (*Penaeus vannamei*). Food Sci. Techn. Internat..

[116] Liao X., Su V., Liu D., Chen S., Hu Y., Ye X., Wang J., Ding T. (2018). Application of atmospheric cold plasma-activated water (PAW) ice for preservation of shrimps (*Metapenaeus ensis*). Food Control.

[117] Kahn M., Lively J.A. (2020). Determination of sulphite and antimicrobial residues in important shrimps to the USA. Aquac. Rep..

[118] Rahman M. Are Ghost Shrimps Edible?. https://acuariopets.com/are-ghost-shrimps-edible/.

[119] Roberts J. Ghost Shrimp (Complete Care, Diet, Set-up and Breeding Guide). http://www.buildyouraquarium.com/ghost-shrimp.

[120] Choi S., Puligundla P., Mok C. (2017). Impact of corona discharge plasma treatment on microbial load and physicochemical and sensory characteristics of semi-dried squid (*Todarodes pacificus*). Food Sci. Biotechnol..

[121] Choi S., Puligundla P., Mok C. (2017). Effect of corona discharge plasma on microbial decontamination of dried squid shreds including physico-chemical and sensory evaluation. LWT-Food Sci. Technol..

[122] Dumen E., Ekici G., Ergin S., Bayrakal G.M. (2020). Presence of Foodborne Pathogens in Seafood and Risk Ranking for Pathogens. Foodborne Pathog. Dis..

[123] Hall A.J., Wikswo M.E., Pringle K., Gould L.H., Parashar U.D. (2014). Vital Signs: Foodborne Norovirus Outbreaks—United States, 2009–2012. Morb. Mortal. Wkly. Rep..

[124] Koopmans M. (2009). Foodborne Viruses and Seafood Safety in an Environmental Health Perspective. Epidemiology.

[125] Mizan F.R., Jahid I.K., Ha S.D. (2015). Microbial biofilms in seafood: A food-hygiene challenge. Food Microbiol..

[126] Sala M.R., Arias C., Dominguez A., Bartolomé R., Muntada J.M. (2008). Foodborne outbreak of gastroenteritis due to Norovirus and *Vibrio parahaemolyticus*. Epidemol. Infect..

[127] Elbashir S., Parveen S., Schwarz J., Rippen T., Jahncke M., DePaola A. (2018). Seafood pathogens and information on antimicrobial resistance: A review. Food Microbiol..

[128] Alfano-Sobsey M., Davies M., Ledford S.I. (2012). Norovirus Outbreak associated with undercooked oysters and secondary household transmission. Epidemiol. Infect..

[129] Brucker R., Bui T., Kwan-Gett T., Stewart L. (2011). Centers for Disease Control and Prevention, Notes from the Field: Norovirus Infections Associated with Frozen Raw Oysters, Washington. Morb. Mortal. Wkly. Rep..

[130] Chironna M., Germinario C., De Medici D., Fiore A., Di Pasquale S., Quartoa M., Barbuti S. (2002). Detection of hepatitis A virus in mussels from different sources marketed in Puglia region (South Italy). Int. J. Food Microbiol..

[131] Centers for Disease Control and Prevention (2013). Increase in Vibrio parahaemolyticus Illnesses Associated with Consumption of Shellfish from Several Atlantic Coast Harvest Areas, United States. https://www.cdc.gov/vibrio/investigations/vibriop-09-13/index.html.

[132] Zuber S.C., Butot S., Baert L., Smulders F.J.M., Nørrung B., Budka H. (2013). Effects of treatments used in food processing on viruses. Food Safety Assurance and Veterinary Public Health, Volume 6: Foodborne Viruses and Prions and Their Significance for Public Health; ECVPH (European College of Veterinary Public Health/European Food Safety Authority).

[133] Choi M.S., Jeon E.B., Kim J., Chou E.H., Lim J.S., Choi J., Ha K.S., Kwon J.Y., Jeong S.H., Park S.Y. (2020). Virucidal Effects of Dielectric Barrier Discharge Plasma on Human Norovirus Infectivity in Fresh Oysters (*Crassostrea gigas*). Foods.

[134] Csadek I., Paulsen P., Weidinger P., Bak K.H., Bauer S., Pilz B., Nowotny N., Smulders F.J.M. (2021). Nitrogen Accumulation in Oyster (*Crassostrea gigas*) Slurry Exposed to Virucidal Cold Atmospheric Plasma Treatment. Life.

[135] van Holde K.E., Miller K.I., Decker H. (2001). Hemocyanins and Invertebrate Evolution. J. Biol. Chem..

[136] Guo L., Xu R., Gou L., Liu Z., Zhao Y., Liu D., Zhang L., Chen H. (2018). Mechanism of Virus Inactivation by Cold Atmospheric-Pressure Plasma and Plasma-Activated Water. Appl. Environ. Microbiol..

[137] Wu Y., Wang Y., Wang J., Xu S., Yu L., Corvini P., Wintgens T. (2016). Nitrate removal from water by new polymeric adsorbent modified with amino and quaternary ammonium groups: Batch and column adsorption study. J. Taiwan Inst. Chem. Eng..

[138] El-Hanache L., Sundermann L., Lebeau B., Toufaily J., Hamieh T., Daou T.J. (2019). Surfactant-modified MFI-type nanozeolites: Super-adsorbents for nitrate removal from contaminated water. Microporous Mesoporous Mater..

[139] Filipić A., Gutierrez-Aguirre I., Primc G., Mozetič M., Dobnik D. (2020). Cold Plasma, a New Hope in the Field of Virus Inactivation. Trends Biotechnol..

[140] McMinn B.R., Ashbolt N.J., Korajkic A. (2017). Bacteriophages as indicators of faecal pollution and enteric virus removal. Lett. Appl. Microbiol..

[141] Cromeans T., Park G.W., Costantini V., Lee D., Wang Q., Farkas T., Lee A., Vinjé J. (2014). Comprehensive comparison of cultivable norovirus surrogates in response to different inactivation and disinfection treatments. Appl. Environ. Microbiol..

[142] Mohamed H., Nayak G., Rendine N., Wigdahl B., Krebs F.C., Bruggeman P.J., Miller V. (2021). Non-thermal plasma as a novel strategy for treating or preventing viral infection and associated disease. Front. Phys..

[143] Weiss M., Daeschlein G., Kramer A., Burchardt M., Brucker S., Wallwiener D., Stope M.B. (2017). Virucide properties of cold atmospheric plasma for future clinical applications. J. Med. Virol..

[144] Mormann S., Dabisch M., Becker B. (2010). Effects of technological processes on the tenacity and inactivation of norovirus genogroup II in experimentally contaminated foods. Appl. Environ. Microbiol..

